# De novo drug design as GPT language modeling: large chemistry models with supervised and reinforcement learning

**DOI:** 10.1007/s10822-024-00559-z

**Published:** 2024-04-22

**Authors:** Gavin Ye

**Affiliations:** Columbia Grammar & Preparatory School, New York, NY USA

**Keywords:** Large language model, GPT, Large chemistry model, De novo drug design, Reinforcement learning, Efficacy optimization

## Abstract

In recent years, generative machine learning algorithms have been successful in designing innovative drug-like molecules. SMILES is a sequence-like language used in most effective drug design models. Due to data’s sequential structure, models such as recurrent neural networks and transformers can design pharmacological compounds with optimized efficacy. Large language models have advanced recently, but their implications on drug design have not yet been explored. Although one study successfully pre-trained a *large chemistry model* (LCM), its application to specific tasks in drug discovery is unknown. In this study, the drug design task is modeled as a causal language modeling problem. Thus, the procedure of reward modeling, supervised fine-tuning, and proximal policy optimization was used to transfer the LCM to drug design, similar to Open AI’s ChatGPT and InstructGPT procedures. By combining the SMILES sequence with chemical descriptors, the novel efficacy evaluation model exceeded its performance compared to previous studies. After proximal policy optimization, the drug design model generated molecules with 99.2% having efficacy pIC_50_ > 7 towards the amyloid precursor protein, with 100% of the generated molecules being valid and novel. This demonstrated the applicability of LCMs in drug discovery, with benefits including less data consumption while fine-tuning. The applicability of LCMs to drug discovery opens the door for larger studies involving reinforcement-learning with human feedback, where chemists provide feedback to LCMs and generate higher-quality molecules. LCMs’ ability to design similar molecules from datasets paves the way for more accessible, non-patented alternatives to drug molecules.

Drug discovery is one of the most time-consuming and costly aspects of developing a drug. It is estimated to take about 10–15 years, with a cost of $1.395 billion per drug discovered and approved [1]. Scientists have attributed the vastness of the chemical space (estimated to have ~10^60^ currently synthesizable molecules) as one of the main challenges in discovering drugs. In simpler terms, it is impossible to enumerate all possible synthesizable molecules to perform virtual screening for drug efficacy. Machine learning (ML) has emerged as one of the most promising tools in drug discovery and can speed up this process. Specifically, scientists have used large datasets of known (drug or drug-like) molecules to train generative machine learning models for designing drug-like molecules with certain desired properties from scratch, or in other words, to perform de novo drug design [2, 3].

There are multiple ways to store these models in a way that a machine can understand, one of which is the simplified molecular-input line-entry system (SMILES). In SMILES, bonds and other geometric information are stored using symbols such as “=” [4]. One can think of it as a chemical language that is used to store the molecule’s structure and its constituents without losing information.

With representation systems that can denote molecules and their structures using concise, one-dimensional sequences, scientists have used ML models such as transformer models and recurrent neural networks (RNNs), which are models commonly used for language processing, for chemistry [3, 5, 6]. This makes these sequence processing models a state-of-the-art approach in de novo drug design. Approaches that uses long short-term memory (LSTM) neural networks and generative adversarial networks (GANs) tended to yield low validity in drug design (i.e., many of the designed molecules are chemically invalid or contain syntax errors) [3, 6, 7]. However, the recent success of large language models (LLMs) and their implications for drug discovery are unexplored. Although there are studies like Regression Transformers [8] and ChemGPT [9] that have successfully adapted large generative pretrained transformers (GPTs) into general LCMs, their roles in drug design are still unexplored. Specifically, one reserach [9] examined model scaling behavior in the context of molecule modeling: the study created ChemGPT, which has successfully transformed large language models into pretrained large chemistry models, generating valid SELFIES molecules [9]. Their study concluded that the *neural scaling* (the trend of drastic increases in model performance as model size increases over several orders of magnitude) phenomenon from NLP also transfers or appears in the field of molecule modeling [9], potentially implicating further breakthroughs. Since ChemGPT was not further trained for specific chemical tasks, the effect of neural scaling in drug design or other specific chemical applications are unknown. In the present study, a LLM (now a LCM) is adapted (trained) to drug design for molecules with high *drug efficacy* for the first time. For case study, the LCM is used to target the amyloid beta precursor protein, a known drug target for Alzheimer’s disease [10, 11]. APP is a drug target in treating AD, as successful inhibition of APP from accumulating amyloid-β peptide could prevent the pathogenesis of AD [10, 11].

The approach used here can easily be adapted to other target proteins. The proposed drug design model can be more data efficient, since LLMs are “few-shot” learners [12]. In other words, the LCM can adapt to specific drug designing tasks with less training data once it is trained for drug design for the first time.

For the first time, the present study uses a drug efficacy evaluation deep learning model that processes both chemical property descriptors and (sequence-denoted) chemical structure by using both recurrent neural networks and dense feed-forward layers. The sequential representations used are SMILES and self-referencing embedded strings (SELFIES). Deep learning refers to the family of artificial neural network models that have multiple layers, which includes traditional feed-forward networks and recurrent neural networks. My study uses this in silico method for evaluating drug efficacy towards the drug target, amyloid-precursor protein (APP). This efficacy evaluation model is later used with my drug design model in a feedback loop for optimizing properties such as drug efficacy via a reinforcement learning optimization technique known as *proximal policy optimization* (PPO).

## Methods

### Method overview

**Fig. 1:**
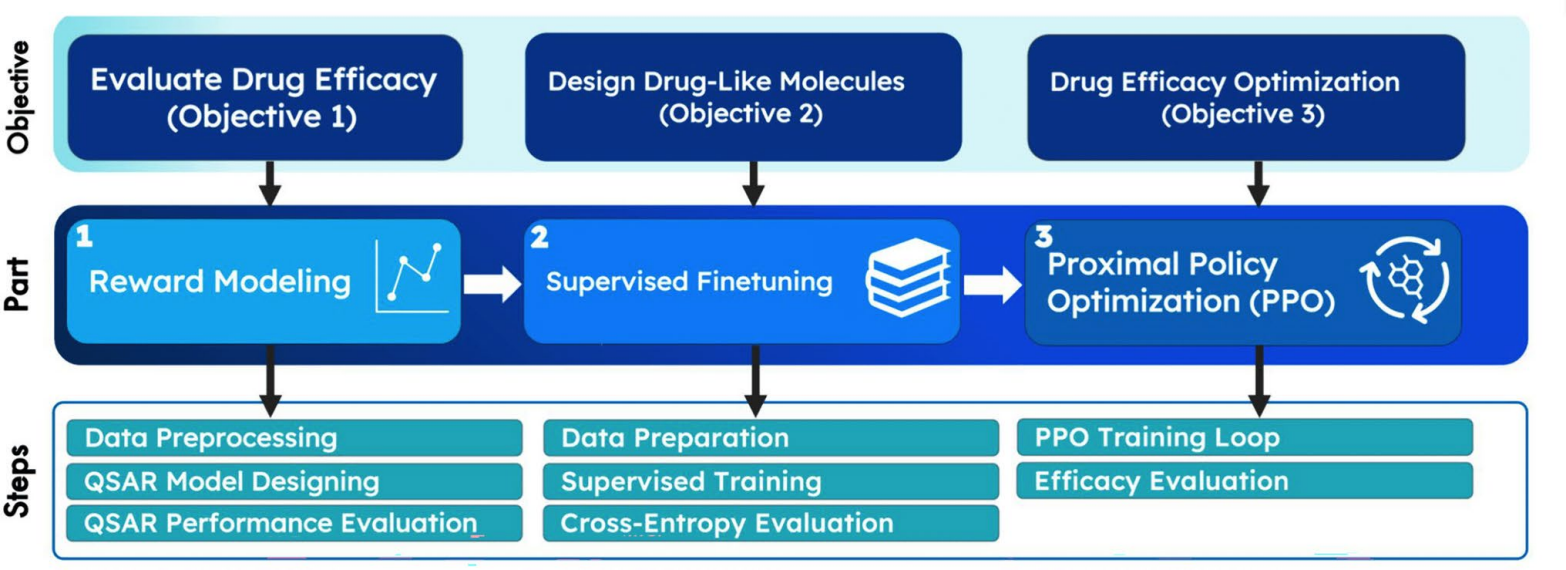
Method overview*.* The steps are listed in chronological order and in which they appear in the paper. The black arrows denote the level of specificity, and the white arrows denote the chronological order. The first part is to train an evaluation model that estimates the drug efficacy given the structure of the molecule. The second part is to train the drug design GPT model to learn how to generate similar drug-like molecules. The third step is to optimize for desired properties such as drug efficacy

The method is organized into three parts: Part 1 addresses Objective 1, Part 2 addresses Objective 2, and so forth (Fig. 1). As stated in the objectives of this study, one of the main goals was to transfer LLM into LCM and adapt LCM for drug design. To achieve Objective 1 in evaluating drug efficacy, I employed the reward modeling step, in which a novel quantitative structure–activity relationship (QSAR) efficacy evaluation model structure was used. To further finetune my drug design model in generating similar drug-like molecules, I used the supervised finetuning (SFT) step. Finally, to use my efficacy evaluation model to optimize my drug design model for designing higher efficacy molecules (also known as developmental candidates), I employed a transformer reinforcement learning (TRL) step by using the proximal policy optimization (PPO) algorithm. The three-step training approach used here is similar to a popular Open AI training approach for training LLMs for ChatGPT. This investigates whether the natural language processing (NLP) training and finetuning schema can transfer for designing effective drug molecules.

For drug design and efficacy optimization, the drug target protein selected is APP, a target that has therapeutic effects in treating Alzheimer’s [10, 11]. However, the same methodology can be applied to transfer LCM for targeting a different protein using a different dataset.

## QSAR evaluation model using LSTMs

To address Objective 1 and to better model drug efficacy towards APP, I devised a novel LSTM QSAR evaluation model design. My evaluation model takes both the structural information denoted in sequence and the chemical properties of the molecule denoted via numerical descriptors. To investigate the explainability of my evaluation model, I used the Exmol library for the first time for efficacy evaluation for aiding ML drug design.

### Data preprocessing

I selected the publicly available APP dataset from BindingDB [13] as the training and evaluation sets. It contains experimental data on ligand–protein interactions (with binding affinity measured, and drug efficacy measured in IC50). For easier processing and better performance of the model, I converted IC50 values to pIC50 values. IC50 measures how much concentration of the drug is needed to have a 50% effect on the biological and biochemical function of the receptor protein. pIC50 can be calculated by using the equation, $pIC_{50} = - log_{10} \left[ {IC_{50} } \right]$, which is a better approach due to easier interpretability and easier processing (not having to deal with extremely small and large floating-point numbers). The higher the pIC50 value, the higher the drug efficacy.

To ensure the uniqueness of the SMILES of the ligands and to avoid discrepancies between the drug efficacy of the same ligand in the data, I used the arithmetic mean of a molecule’s multiple recorded pIC_50_ values, as now each molecule corresponds to one drug efficacy value only. Since most of the measurements in the dataset are in pIC_50_ (converted from IC_50_), I filtered and used the experimental data measured in pIC_50_, leaving 1032 unique experimentally tested ligands in total, some of which are patented molecules. I later split the dataset into 80% for training and 20% for evaluation.

### Alternative sequential representations

To investigate the most suitable sequential representation of molecules for my efficacy evaluation model, I trained evaluation models using SMILES and SELFIES. There are many different ways to encode SMILES into tensors to feed them into the neural network QSAR model. The most common and straightforward approach is to use a custom Pytorch embedding layer, and map each character of the sequence into an unique integer. The embedding layer is a neural network layer whose goal is to help learn the semantic relationships between tokens. Semantic relationships can be, for instance, that “(” are always paired with “)”, and “c” is the same as “C” but part of an aromatic ring. To map each character to integer, thus turning the whole sequence into a tensor, I modified and used the vocabulary list from Abbasi’s study [3], which contains an almost complete list of valid characters in SMILES. Alternatively, instead of SMILES representation, one can also use SELFIES, which requires no additional data preprocessing since it is already in tokenized format. Thus, although SMILES can be sometimes more concise, SELFIES can be more “machine-friendly,” which is why I compared QSAR evaluation models trained using SMILES representation and those trained using SELFIES representation.

To select the best way to process sequential information for my evaluation model, I used the DeepChem’s Mol2Vec embedding system [14]. Mol2Vec [14] is a pretrained embedding layer network on PubChem database’s molecules [15]; it is the analog of the Word2Vec embedding network—a commonly used pretrained embedding system from NLP. I tested these 3 different embedding techniques and selected the more suitable, best performing representation and embedding system for the final efficacy evaluation model.

### Feature engineering and QSAR evaluation model designing

**Fig. 2:**
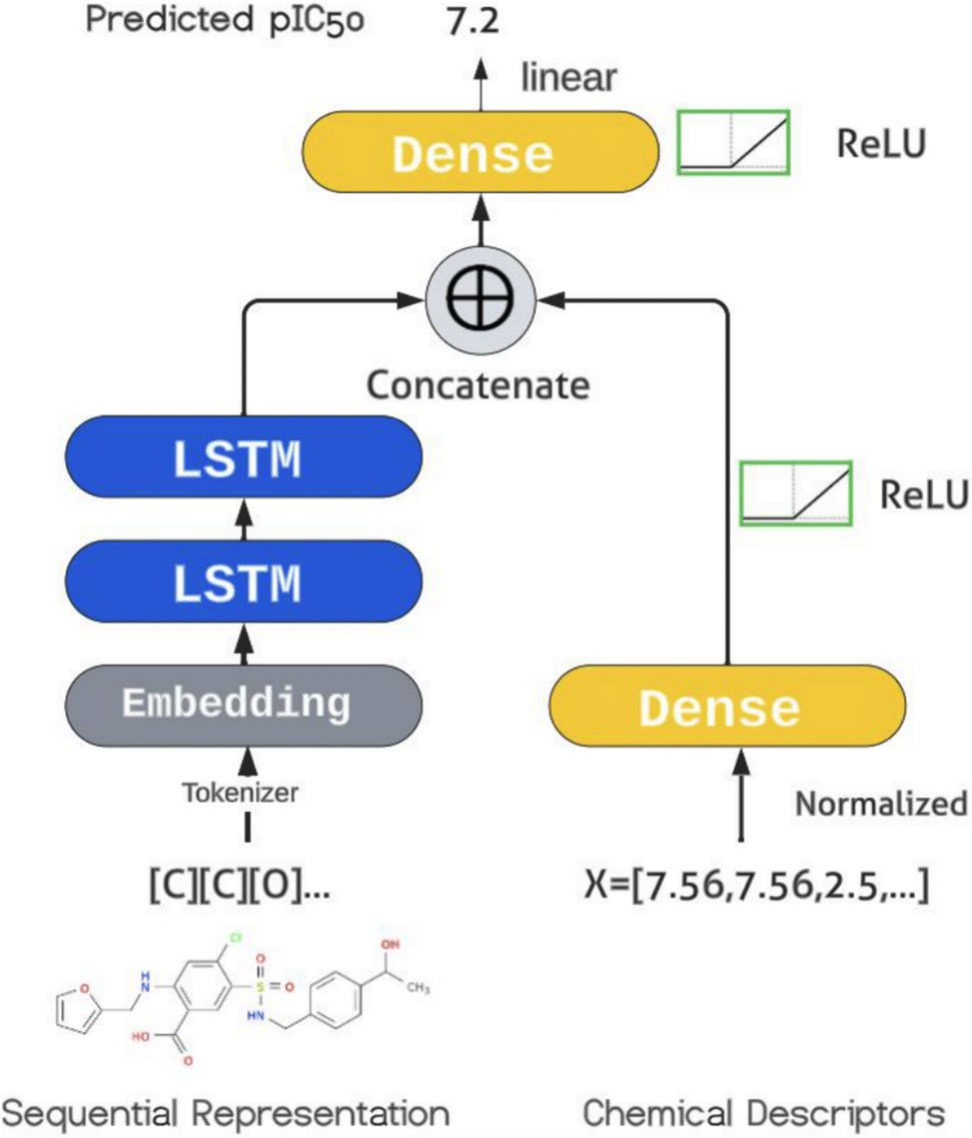
QSAR evaluation model: multi-feature LSTM architecture. The QSAR architecture takes in two different representations of a molecule: in sequential representation using SELFIES, and in chemical descriptors generated by RDKit. The two inputs go through different neural network layers, then the output of the layers are concatenated and go through one final layer to produce the predicted pIC_50_ value. For the embedding layer, a custom embedding layer and DeepChem embedding layers were tested

I designed the QSAR efficacy evaluation model, which is novel since it takes in both sequential representation and numerical chemical descriptors of molecules calculated from RDKit [16] to achieve better performance compared to previous models (shown in Fig. 2). RDKit Tools is a python library that has helper functions such as to calculate chemical descriptors or to parse and validate SMILES. The numerical chemical descriptors from RDKit were calculated and used as features, in addition to the sequential representation input feature (in SMILES or SELFIES). These chemical descriptors are numbers representing the molecule’s chemical properties, such as the polar surface area, etc. Numerical descriptors have shown to be useful for QSAR, even outperforming one-hot-encoded structure representation when non-sequential processing models are used such as support vector regression and random forest [17]. I filtered out the non-numerical descriptors from RDKit and used DeepChem’s RDKit descriptors for QSAR. I normalized the descriptors via scikit-learn scaler (normalizer) since different RDKit descriptors span in different scales. Features with drastically different scales can be hard to learn for neural networks.

To train my evaluation model, I searched for the optimal hyperparameters via grid search. The tuned, final hyperparameters for the LSTM (evaluation) model used a learning rate of 0.001 and a dropout of 0.3 to prevent overfitting. The embedding layer supports 701 different SELFIES encoded tokens. The learning rate controls how much the model changes when making a mistake, and the dropout prevents overfitting by training only a certain percentage of randomly picked neurons at a time. The loss function is defined as the mean squared error (MSE) loss, a common loss metric for regression.

I trained the evaluation model with a batch size of 16. The model itself takes in two inputs, the first one being a sequence of token IDs where each id is an integer denoting a token, the second input being a feature vector of numerical, RDKit generated chemical descriptors of the molecule. In my evaluation model (Fig. 2), each layer consists of 256 neurons. Inside the model, the first, sequential input is passed into the embedding layer, and the second, numerical, input is passed into a dense layer directly. Then, the output of the embedding layer is passed into two layers of LSTM, and the output of the two LSTM layers are concatenated with the output of the first dense layer, into one final feature vector, which is fed into a dense, feed-forward layer, whose output is finally fed into one neuron in the next layer and the predicted value is outputted from this neuron (Fig. 2). The number of dense layers processing the chemical descriptors were tuned, and the hyperparameters were tuned. The activation function used for the layers was the rectified linear unit (ReLU).

To train and compare my QSAR evaluation model that uses Mol2Vec embedding system [14] and my model that uses SMILES representation, I used the same model structure, except only 82 tokens (excluding default ones such as the end of sequence token) were used for the embedding layer for the evaluation model that uses pure SMILES. During training, I used the Adam optimizer with betas. They were used to average the gradient of previous *n* batches, which has been shown helpful for models’ lost functions to cross through saddle points and local minimas. To decrease the learning rate when the model was converging (and when the validation metrics were not improving), I used the Reduce Learning Rate on Plateau scheduler, making the training more stable. I trained the evaluation model for 100 epochs, and I selected the best performing model as the final efficacy evaluation model.

### QSAR performance evaluation

To better measure the performance of my evaluation model in addition to the loss metric, I used the concordance correlation coefficient (CCC) as it is a better metric for both bias and variance (i.e., for both “accuracy” and “precision”). To explore how efficiently my evaluation model uses its data compared to previous models, I conducted another experiment with the same procedure except the training sets used have size [825, 700, 500, 400, 250, 100]. Since the dataset only had 1032 unique SMILES, the maximum size used was $825$ molecules for training (using a 80–20 train-test data split). I shuffled the dataset and split into training and evaluation once; I used a portion of the training set to train the evaluation model. The evaluation set stayed unchanged during the experiment. Splitting only once throughout this experiment reduces the effect of randomness on performance since each time the trained model is evaluated on the same set of molecules.

### Model explainability

To open and take “sneak peeks” into the black box of this LSTM neural network in order to provide explainability to my evaluation model, I used the Exmol python library. It is hard to interpret neural networks since they are black boxes. Unlike decision tree-based models, which are self-explanatory with its branch denoting a specific condition of some feature, a neural network has so many parameters, weights, and biases that it is impossible for any human to investigate why and what patterns and strategies did the evaluation model learned; this is known as the black box phenomenon: input and output can be observed, but not anything that happens in between. Exmol is a python library that uses different chemical descriptors and surrogate models to explain a chemistry related regression or classification model [18]. This is a relatively new tool as the library is released in late 2022. This is the first time it is used for aiding ML de novo drug design by making drug efficacy evaluation model interpretable. I used the default Exmol descriptors, and the results were automatically graphed by the library. A natural language explanation can also be generated, which explains what is happening behind the evaluation model [18]. Furthermore, I compared the Exmol descriptors with findings from previous studies on drug efficacy and activity to determine the credibility of using Exmol. If the result proves credible, then Exmol serves as a convincing way of interpreting QSAR neural networks. If credible, explainability of QSAR efficacy evaluation models can provide invaluable information for human chemists on what makes a ligand effective towards the drug target.

## LCMs for adapting to drug discovery

In my study, I treat the problem of drug design as a *causal language modeling* (CLM) problem, like many other GPT models in NLP. In CLM, during training, the model tries to predict the next word in the sequence in some dataset, and then in generation, it generates new sequences word-by-word and token-by-token.

To perform causal language modeling for supervised finetuning, in addition to using Pytorch, I used the Hugging Face transformers library and the Hugging Face transformer reinforcement learning (TRL) library. Hugging Face is a website and a community that has public, open-source repositories of large language models, such as Meta’s Llama2 [19], and Falcon LLM [20]. This addresses Objective 2, to train my drug design model to design similar drug-like molecules, adapting the model to drug design from general chemical tasks.

The Hugging Face transformers library provides many helper functions needed to train one’s own transformer. The transformer reinforcement learning (TRL) library has functions and classes that allow customized reinforcement learning training. I used TRL since it can easily interact with Hugging Face causal language models and contains state-of-the-art reinforcement learning algorithms that are commonly used in the field of natural language processing.

### Supervised finetuning of LCM

The base transformer model used was the GPT-Neo language model [21], pretrained by the study on ChemGPT [9] using 10 million molecules from PubChem [15] to generate general molecules. To adapt the LCM to the APP dataset, I devised this supervised finetuning step to train the LCM.

### Data preparation

To perform supervised finetuning, I used the same BindingDB dataset [13] from the QSAR training. I used the same dataset cleaning technique, and the dataset was also mapped from tokenized SELFIES into numerical token identifiers (token IDs) using a modified version based on ChemGPT’s auto tokenizer from Hugging Face library. The tokenizer was updated to support all of the tokens used in the dataset, and thus the embedding layer of my drug design model was resized. These new tokens include chirality information, such as the “[C@Hexpl]” and “[C@@expl]” SELFIES encoded tokens. Similar to the procedures for QSAR, I split the dataset into 80% for training and 20% for evaluation.

After the tokenizer was prepared and the training dataset was tokenized, to investigate whether chunking (one NLP data processing technique) helps LCM performance, I compared LCMs that were tuned with and without this chunking preprocessing. Chunking refers to merging and splitting multiple input sequences together to convert all input sequences into chunks having the same length. Each chunk is an incomplete sequence, with tokens representing the start and the end of an individual sequence respectively. In other words, after chunking, many of the chunk sequences will not contain both the start token and the end token unless the chunk contains a complete sequence. This is done because transformers have a limited context window (can focus and process a certain amount of words at a time), and it is more efficient for the transformer model to utilize the full window during training. However, natural language modeling models are usually trained via documents and encyclopedia articles that are far longer than the context window length. In contrast, for drug design, all of the ligands from the dataset have a length less than 200 when represented in SELFIES encoded tokens; all are smaller than the window length. Thus, I trained the drug design model with and without this technique and compared the loss values to evaluate whether the chunking technique affected the performance, or if chunking caused the drug design model to incorrectly assume relationships between the independent short SELFIES sequences. I chose the better performing supervised-trained drug design model for Part 3 of the method.

### Supervised training

Finally, after data preparation, I used the supervised finetuning trainer from Hugging Face to configure and train the drug design LCM. The trainer automatically batched input (token ID) sequences and pad them into the same length behind the scene, thus no further data preprocessing was needed. I used these padding tokens to account for the fact that different sequences have different lengths, while the input tensor must be rectangular—each sequence having the same length. In addition, I also used the attention masks to tell the transformer model to ignore padding tokens. I used a batch size of 16, and the learning rate was set to $7\cdot 1{0}^{-5}$. I used a weight decay of 0.01 to prevent overfitting and trained the drug design model for 10 epochs. I tuned the hyperparameters via Optuna [22], a Python Bayesian parameter optimizer, which was used since it is compatible with Hugging Face, and the Bayesian parameter optimizer adapts to the performance of drug design models with different hyperparameters rather than simply enumerating through a grid of combinations of hyperparameters. This can be relevant and important when the training process is relatively time consuming.

### Model evaluation using cross-entropy loss

To train the drug design LCM via supervised finetuning, I used the loss function, which is defined as the cross-entropy loss (Eq. 1):

1 $$ L\left( {p,q} \right) = - \sum\limits_{x \in vocabs} {p\left( x \right)log\left( {q\left( x \right)} \right)} $$

where $L$ represents cross-entropy loss, which takes in the true next token, $p$, as a probability distribution where the correct token has a 100% probability and other tokens having a 0% probability, and the model’s distribution (which is the logit) is denoted as $q$. I used the cross-entropy loss to define how close the model’s logit is from the true distribution, so the gradient can be computed and the model can be corrected. In causal language modeling, when generating each token, the cross-entropy loss was used for correcting a model when its response deviates from the sequence or molecule it is trying to model. In other words, the gradient is computed and the model learns from errors it makes anytime it generates a token that differs from the token from the “correct answer” sequence, which is the desired next token.

To evaluate my drug design model’s performance after the training was complete, I repeatedly prompted my model with the start token for performing de novo drug design. I graphed and compared the distribution of drug efficacy, and other chemical properties of the generated molecules with the dataset molecules. The validity and novelty of the generated molecules were also determined. Validity is defined as the percentage of generated sequences that are parasable to represent actual molecules; novelty is defined as the percentage of generated sequences that do not exist in the dataset (both training and evaluation set). This supervised finetuning trained drug design model serves as the basis model for PPO training in the next step.

## Proximal policy optimization (PPO)

The role of PPO is to incentivize my drug design model for designing higher efficacy molecules. To the best of my knowledge, no published study on machine learning aided drug design has used PPO.

### Reinforcement learning notations

To use reinforcement learning for drug design LCM, one will need to define each component of the reinforcement learning (RL) agent. In this case, the LCM, or LLM, is the policy function of an agent (denoted as *π*_*θ*_* (a*_*t*_*|s*_*t*_*)*), where each action of the agent is to generate the next token, and the state *s*_*t*_ represents some already generated sequence (recall the CLM procedure). The advantage function, or *A(s*_*t*_*,a*_*t*_*)* or *A*_*t*_ represents how good or bad some decision* a*_*t*_ is. It can be described mathematically as *A*_*t*_ = *Q(s*_t_*,a*_*t*_*)−V(s*_*t*_*)*, where *Q(s,a)* represents the expected desiredness of performing some action *a* at some state, subtracted from *V(s)*, the desirability of this current state; thus, the whole term determines how much more desiredness or advantage one get by performing this action. To define and incentivize for “desired, effective molecules” using RL, one method is to take the gradient with respect to the policy’s parameters that optimizes the advantage function, which is one of the main ideas behind the proximal policy optimization algorithm*.*

### The value head

The purpose of the value head is to make the LCM drug design model prefer a higher reward given by designing desired, effective molecules. During reinforcement learning, the value head is connected to the generative LCM model, which is an additional feed-forward neural network that represents the value function. This is the part of the model that “communicates” with the efficacy evaluation model to interpret the evaluated efficacy “score”.

### PPO definition for drug design & implementation details

To incentivize the drug design LCM to generate higher efficacy molecules towards APP, I used a PPO reinforcement learning schema. To do so, I used the efficacy evaluation model trained earlier for scoring molecules, designed by the drug design LCM trained from Part 2. I repeated the training loop multiple times throughout the algorithm for multiple epochs.

Through reinforcement learning, the drug design model finds out what sequences of tokens work well (have high drug efficacy) and what does not. I used the PPO Trainer from Hugging Face for this training process, which updates the drug design model behind the scene when given the three parameters: $\left( {Q = \text{query} = \text{input},A = \text{generated},R = \text{reward} = \text{drug efficacy}} \right)$. The PPO algorithm was implemented in the trainer’s step function, and the training loop was designed and implemented by myself. The present study should be the first time that reinforcement learning optimization (specifically PPO) is applied to LCMs.

Formally, the PPO algorithm can be described as maximizing the following equation, for some hyperparameter epsilon denoting how much a policy is allowed to change each update, shown in (4):

2 $$ Objective^{PPO} \left( \theta \right) = \hat{E}_{t} \left[ {r_{\pi } \left( \theta \right) \cdot \hat{A}_{t} } \right] $$

where $$r_{\pi } \left( \theta \right) = clip\left( {\frac{{\pi_{\theta } (a_{t} |s_{t} )}}{{\pi_{{ \theta_{old} }} (a_{t} |s_{t} )}},1 \pm \varepsilon } \right)$$.

Where $Objective^{PPO }$ represents the objective of PPO, *s*_t_ represents the expected value at some time *t*, and the conventional notations for RL are used.

To make the training more stable, PPO limits the amount of changes possible per training step, minimizing the consequence of an inaccurate value-head (*V(s)*). Since the advantage term ${\widehat{A}}_{t}$ depends on what a model estimates how good an action is at some state (or *Q(s,a)*), it depends on the value function, which is an approximation made by the value head of the policy network. To make the training more stable, one needs to limit the amount of change it can perform in a single update to prevent an initially inaccurate value head from misleading the policy network, by setting a maximum threshold, for instance. This technique is called “clipping.” KL divergence is a similar popular technique that can be used for limiting the amount of change compared to the old policy in PPO training. Simply put, KL divergence takes in the new and old policy distribution (the probability of taking action a for all actions) and outputs a nonzero real number—the larger the output the larger the difference. Formally, it is defined as in (3):

3 $$ D_{KL} (P||Q) = \sum\limits_{i} {P\left( i \right)log\left[ {\frac{P\left( i \right)}{{Q\left( i \right)}}} \right]} $$

where the $P$ and $Q$ distributions are the policies, and $i$ is any action that the policy can take. In the drug designing case, each action corresponds to generating some unique token IDs representing some information of the designed molecule. The policies are the drug design LCMs.

### PPO training loop

The initial PPO policy network in this case is the supervised finetuned drug design LCM. I employed the default KL-divergence penalty to ensure my drug design model did not deviate too much away from the original supervised finetuning trained model.

**Fig. 3:**
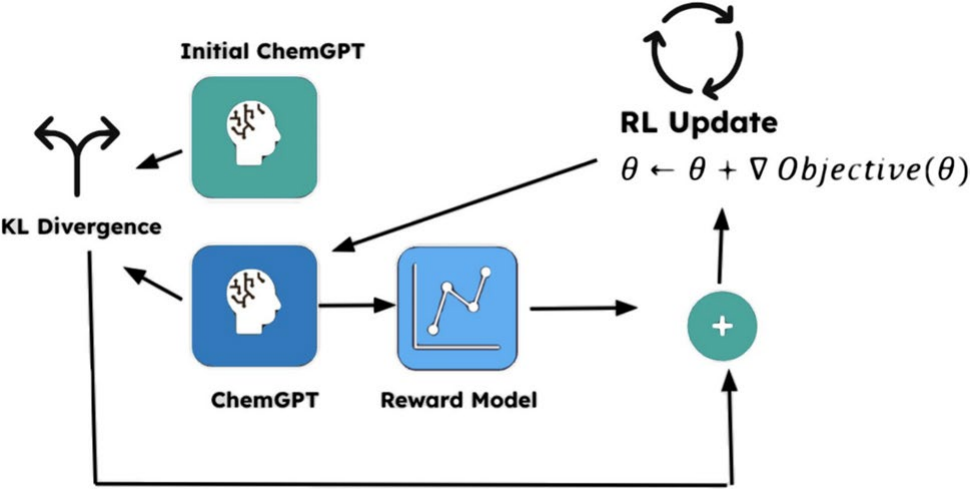
Feedback loop of proximal policy optimization*.* The PPO training loop is summarized in the diagram. In a nutshell, the designed molecules of my drug design model (shown as “ChemGPT”) are scored by the reward (evaluation) model. Each score is then combined with the KL divergence for more stable model performance and to ensure model convergence. My initial drug design model served as a constraint to limit deviation of my updated drug design model. The combined value is used to update the drug design model’s parameters by taking the gradient of the objective (with respect to $\theta$, the parameters). Notice that the “( +)” means combined since the reward model outputs a reward while the KL divergence outputs a penalty (-reward). This training loop is repeated multiple times to maximize the reward received by the model

For training, I trained the drug design LCM for 14 epochs, with a learning rate set to 1.41. For this training, I used the same training set that was used for supervised finetuning from Part 2, except, some of the molecules were not used for generation at all and were replaced with the start token for generating from scratch; molecules that were used only had the first 2–8 tokens to guide the drug design model during PPO. This is a common technique in finetuning LLM when using PPO. The training loop (Fig. 3) created can be broken down into three parts: action (generation), reward prediction, and optimization.

During generation, I set the top p value to 1.0 (or 100%), encouraging the generator to explore more, rather than to only exploit what it already knows to maximize reward. In other words, top p = 1.0 allows the drug design LCM to consider all possible actions, with better actions having higher probability to be chosen. In contrast to greedy strategy (top k = 1), which chooses the token that it *currently* estimates is the most optimal, this top p = 1 policy does not have early suboptimal convergence, since one of the pitfalls of greedy strategy is that the locally best choice of action isn’t always the best choice overall. The drug design LCM generates a list of token IDs sequences, each representing the SELFIES encoded sequence of the designed molecule.

In the second step, my code from the training loop decoded these generated sequences of token IDs into SMILES sequences, which were then parsed and calculated using RDKit for their chemical descriptors. With the original generated sequences padded and the chemical descriptors ready, I used the trained QSAR evaluation model for estimating the reward received (the efficacy) for each designed molecule.

Finally, my training loop passed the input, the generated token IDs, and the rewards into its PPO train step function for running the PPO algorithm for updating the policy network (my drug design model). I repeated this 3-part process for each batch from each epoch until the PPO training was complete.

### PPO evaluation

To evaluate the extent to which the post-PPO LCM designed high efficacy molecules, I graphed and compared a kernel density estimation distribution of drug efficacy (pIC_50_) of generated molecules with the drug efficacy distribution of the dataset molecules.

## Results & discussion

One of the main goals of this study was to transfer a LLM into a large chemistry model (LCM) and adapt LCM for drug design for the first time. The successful application of LLM (or LCM) and NLP training schemas in drug discovery in this study is novel, and the application of reinforcement learning on LCM to drug design is a strategy that had not been investigated in previous studies. In the present study, I chose APP for the case study of drug design, and the same LCM can be transferred to target a new protein utilizing a different dataset using the same methodology. This study used a three-step NLP strategy, which used a PPO algorithm with my QSAR efficacy evaluation model to optimize the drug efficacy of the molecules designed by my drug design LCM. Furthermore, this study employed an unexplored approach for QSAR using a combination of sequential and numerical chemical descriptors, which achieved a better performance. Then, the Exmol library was used as a way to interpret my QSAR efficacy evaluation model. This utilization of Exmol library on pIC50 efficacy for ML drug design represents a previously unexplored approach.

## The novel QSAR model outperforms traditional QSAR models

**Fig. 4:**
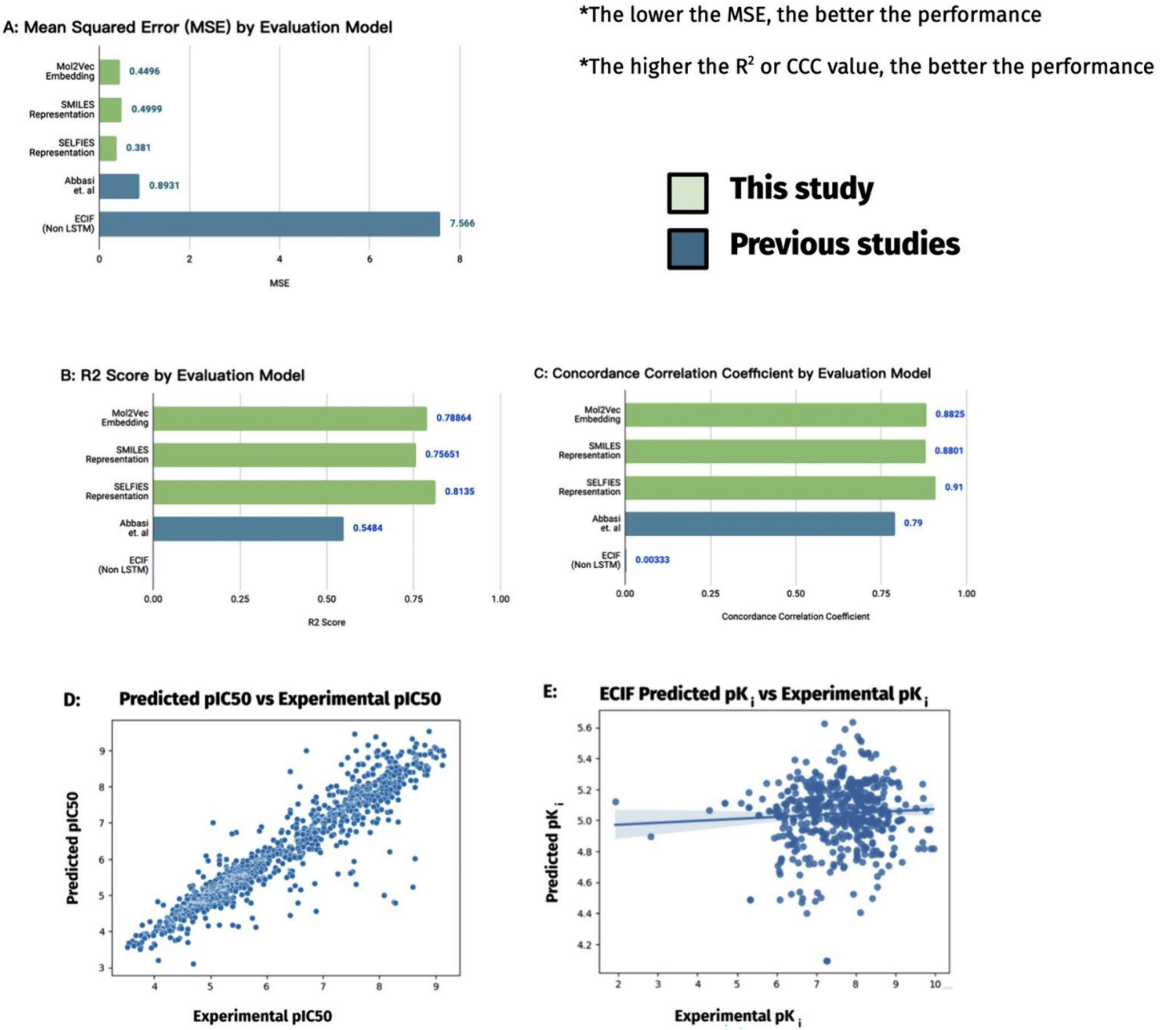
Performance of QSAR efficacy evaluation model exceeds previous studies model performance. **A–C** the performance of each model on the testing set over three different metrics. The models tested are Abbasi et al.’s LSTM model [3], the ECIF random forest model [23], and my models trained with three different molecule representation formats. MSE, or mean squared error, is a metric to measure the overall difference between experimental and predicted pIC_50_ values, punishing larger differences more due to the squared terms. The green highlighted bars represent models from the present study, and the blue highlighted bars are from previous studies. **D–E** compares the performance of the QSAR model over the entire BindingDB dataset (on the left) vs. the performance of the ECIF random forest model. (on the right). The regression line on the right is much more flat (which means a weaker performance) than the regression line on the left, which is consistent from findings of previous study that sequential QSAR LSTM models outperform previous QSAR models [3]. K_i_ is an alternative way of measuring efficacy, which was used since the ECIF model outputs pK_i_ values—although efficacy was measured in different metrics, both are from the same BindingDB dataset

To better model drug efficacy for generated molecules and to later use it for efficacy optimization, I devised and used the QSAR evaluation model that takes a combination of sequential and chemical descriptor data for the first time. The performance of my evaluation model on the experimental data was evaluated and compared with prior QSAR models (on the same dataset): the LSTM model from Abbasi et al.’s study [3] and the ECIF random forest model [23] (see Fig. 4). The result showed that the addition of chemical descriptors increased the model performance (concordance correlation coefficient (CCC) of 0.91 vs. 0.79), being 2.34 times better in performance (by a 2.34 times smaller MSE loss) compared to previous study’s LSTM model.

Having a much more accurate reward (evaluation) model is important as it guides the generative drug design model in PPO training towards designing higher efficacy molecules by scoring. Thus, my evaluation model with SELFIES-encoded sequence input was chosen as the reward model. Although the SELFIES-based and SMILES sequence-based models had relatively similar performance, the SELFIES-based model can more easily interact with my drug design model, which generates molecules in SELFIES tokens. A possible explanation for this result is that with the addition of numerical descriptors, my evaluation model no longer needs to rediscover how to estimate these descriptors or patterns (such as the amount of hydrogen bonding) themselves using the sequence and can focus on other aspects of the structure provided by the sequence. The results demonstrates that Objective 1 was achieved successfully: my QSAR evaluation models with combined sequence and numerical descriptor input indeed achieve better performance than traditional structure-only QSAR models.

The increased performance in the evaluation model makes computational drug discovery more reliable (for APP in this case study). The drug discovery process is accelerated by this more than twofold improvement in performance, as a more accurate evaluation model reduces the failure rate of designed effective molecules and is therefore more likely to hold true during actual in-vitro experimental validations.

### Novel QSAR structure is data-efficient

To explore how efficiently my QSAR evaluation model uses its data compared to previous models, another experiment was conducted with different sizes of training sets.

**Fig. 5:**
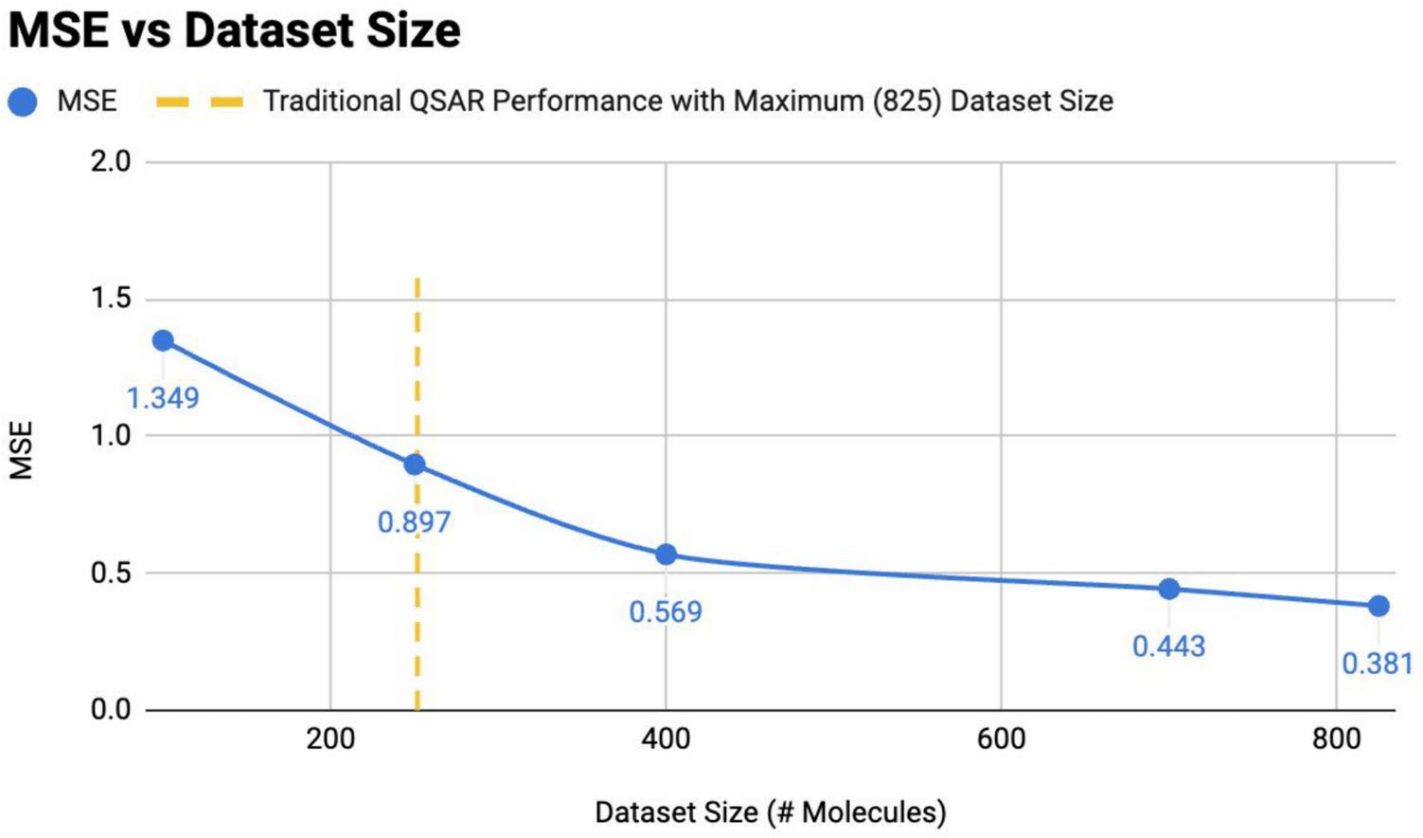
Novel QSAR model maintains outperformance as dataset size decreased. Mean squared error (MSE) values are used in the figure as a metric for model performance, where the lower the MSE value, the better the performance. The QSAR (evaluation) model designed in this study was trained with different sizes of datasets. The evaluation dataset used remained unchanged throughout the experiment. The rightmost three values are ones that are smaller than the MSE value for a traditional LSTM QSAR model [3] trained using the same 825 molecules dataset. The loss of the QSAR model increases as the training dataset size decreases; however the QSAR model still performed better, until the dataset size became around 250

A size-difference of around 150 datapoints was used to estimate the performance of my final evaluation model over each dataset since enumerating all possible dataset sizes can be time consuming. The result from Fig. 5 implies that my SELFIES-based evaluation model, using 70% less data, achieves similar performance compared to traditional LSTM QSAR regressors (250 dataset molecules vs. 825 molecules). This implies that the addition of chemical descriptors does indeed decrease the amount of data required for the model to learn through the structural relationships. This also demonstrated that my evaluation model structure in the present study performs better even when information is more limited (fewer experimental data available), making it applicable in earlier stages of drug discovery, when the disease or the drug target is first discovered.

### Neural network QSAR model can become explainable

**Fig. 6:**
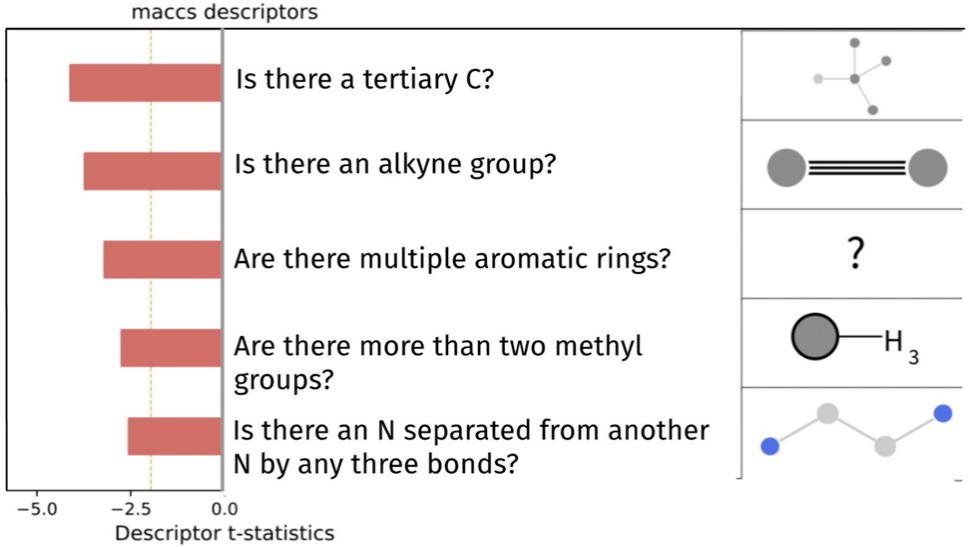
Correlation between descriptor and efficacy by QSAR model demonstrates explainability*.* The Exmol library [18] was used in attempts to make the QSAR model explainable. A molecule was sampled from the training set and different variants of the molecule were produced by the Exmol and the relative increase or decrease in predicted efficacy was recorded. The sign of the t-value represents negative or positive correlation, while the magnitude represents the statistical significance. The yellow dotted line represent statistical significance threshold, and the red bars represent negative correlation. All of the top 5 default molecule descriptors are statistically significant

To retrieve valuable information on what makes a molecule effective, my QSAR efficacy evaluation model must be explainable. To attempt to explain the QSAR evaluation model, which is a neural network black box, the Exmol python library was used for efficacy for the first time: Exmol uses molecule descriptors and surrogate models to record correlation between molecule descriptor and predicted activity by querying different variants of a molecule (shown in Fig. 6). The results show that having a tertiary carbon hinders a molecule’s efficacy towards APP, so does an alkyne group, too many aromatic rings, too many methyl groups, or largely separated nitrogen.

Previous studies had shown that having an excessive amount of aromatic rings (more than 3) is negatively correlated with the druggability and developability of a molecule, regardless of the target protein [24]. Similar to the excessive number of methyl and alkynes groups, the tertiary carbon and separated nitrogen descriptors are more general and no previous studies have proven or disproven their hindrance on APP efficacy of a molecule. One possible explanation is that these correlations only apply to APP only and do not generalize to protein–ligand interactions overall. Future studies can investigate the potential of using more customized, more specific molecule descriptors with Exmol for more detailed information.

Although more customized, specific molecule descriptors for APP can make one retrieve more specific information from these “sneak peeks,” the results suggest that the explainability of QSAR evaluation models can provide credible information for human chemists on what makes a ligand effective towards a drug target. This can accelerate chemists’ understanding of drug targets and effective ligands. In addition, future study can investigate the potential in the information derived from this approach to serve as additional conditions or optimization goals for a drug design model—breaking down an abstract metric into multiple easier goals can potentially increase a drug design model’s performance. Having a better understanding of the essence which makes a molecule effective can be important since although a drug-like molecule can be potentially discarded in later stage, this information can be reused throughout the entire process: for instance, when optimizing the molecule at a later stage such as for lowering toxicity, chemists would know what are essential for the efficacy and must not change while what can be optimized and adjusted.

## Supervised finetuning can help drug design LCM to model drug-like molecules

To better model the molecules from the dataset (to be “drug-like”) before optimizing for any properties, this study used supervised finetuning (SFT) for the LCM to model the molecules from the same BindingDB dataset used for reward modeling. Different configurations with different hyperparameters were tried, and the best model successfully learned to generate similar molecules and the distribution of molecules from the dataset (cross-entropy loss of 0.1253; with chunk preprocessing). Next, the property distribution of 168 sampled generated molecules was calculated and compared with the dataset’s molecules.

**Fig. 7:**
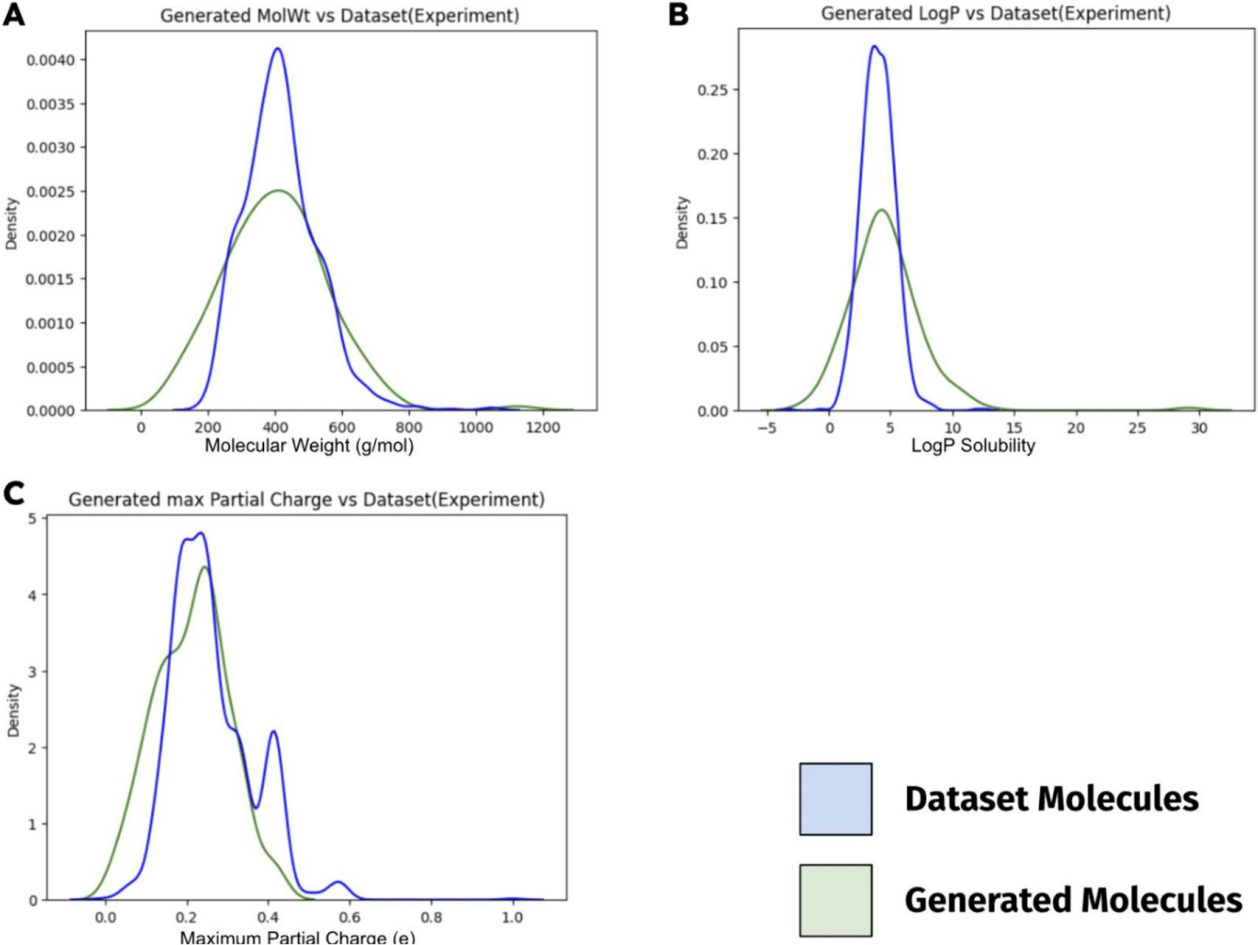
Molecule property distribution of generated is similar to distribution of dataset Molecules. 168 generated molecules’ properties were calculated and graphed. Density is proportional to the frequency, except the graph is normalized, thus sample size is not considered in the comparison. The graphs showed that most generated molecules do lie in the same approximate range as the drug candidates from the dataset in different molecule properties. The difference in the tails of the distributions can be attributed to the difference in the sample sizes: 168 vs. 1024

A kernel density estimation (KDE) distribution graph is a smooth curve that estimates and interpolates the frequency distribution of some continuous data from a set of recorded data points. The KDE distribution plots (Fig. 7) suggest that my drug design model successfully modeled the various property distributions of the molecules from the dataset, further supporting the small loss value.

Having the drug design LCM learn from the distribution of molecules in the dataset is important: ChemGPT is a general pretrained model that has not been tuned for drug-like or even organic molecules. In addition to helping the drug design LCM better converge in the PPO optimization process, the fine-tuned drug design LCM serves as a basis model, preventing the optimizing LCM from deviating and generating invalid and not drug-like molecules. My drug design model’s successful convergence, as portrayed by the figure (Fig. 7), further proved LCM’s ability to use CLM to model drug-like molecules in a supervised setting, as shown in [9].

A potential implication of this is to use LCMs for learning or “deep-faking” patented drug molecules, which can be used by a future study for discovering alternative, more accessible, and similar drug molecules such as for aiding developing regions. This can be useful such as for tuberculosis, where effective drugs exist but are inaccessible in many developing regions due to high cost.

## Proximal policy optimization can optimize efficacy for drug design LCM

**Fig. 8:**
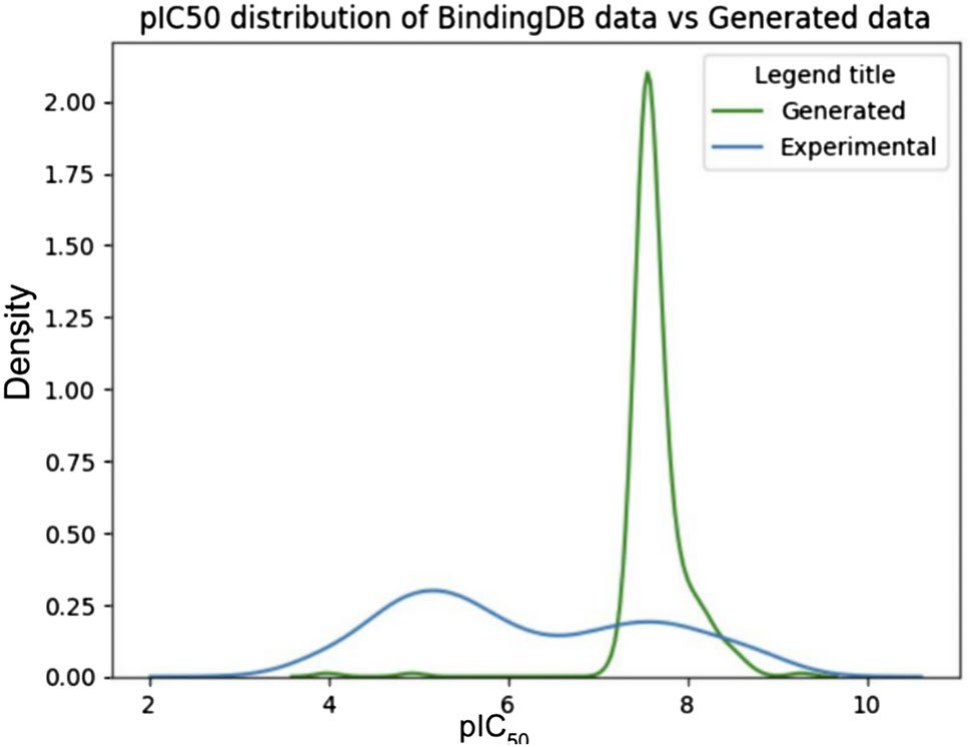
Average Drug Efficacy (pIC_50_) of Generated Molecules Exceeds Dataset Molecules. Two hundred and fifty-six molecules were sampled and their drug efficacy calculated. Density is proportional to the frequency, except the graph is normalized, thus sample size is not considered in the comparison. The peak in the green distribution curve shows that most generated molecules have a drug efficacy around 8, while the blue curve, which represents the dataset molecules, has two smaller peaks, meaning most molecules had a pIC_50_ efficacy value of either around 5 or around 8

To optimize the drug design LCM in generating molecules with high drug efficacy and other properties (the novelty and pIC_50_ are optimized as examples), the PPO algorithm was employed using the reward and SFT-trained drug design models. The KDE distribution of drug efficacy (pIC_50_) of molecules after PPO was graphed and compared with the drug efficacy distribution of the dataset (Fig. 8). My drug design model’s ability to generate desired molecules is shown by a 15.49 times more effective mean efficacy value (in IC_50_) in the generated set of molecules than in the dataset.

**Fig. 9:**
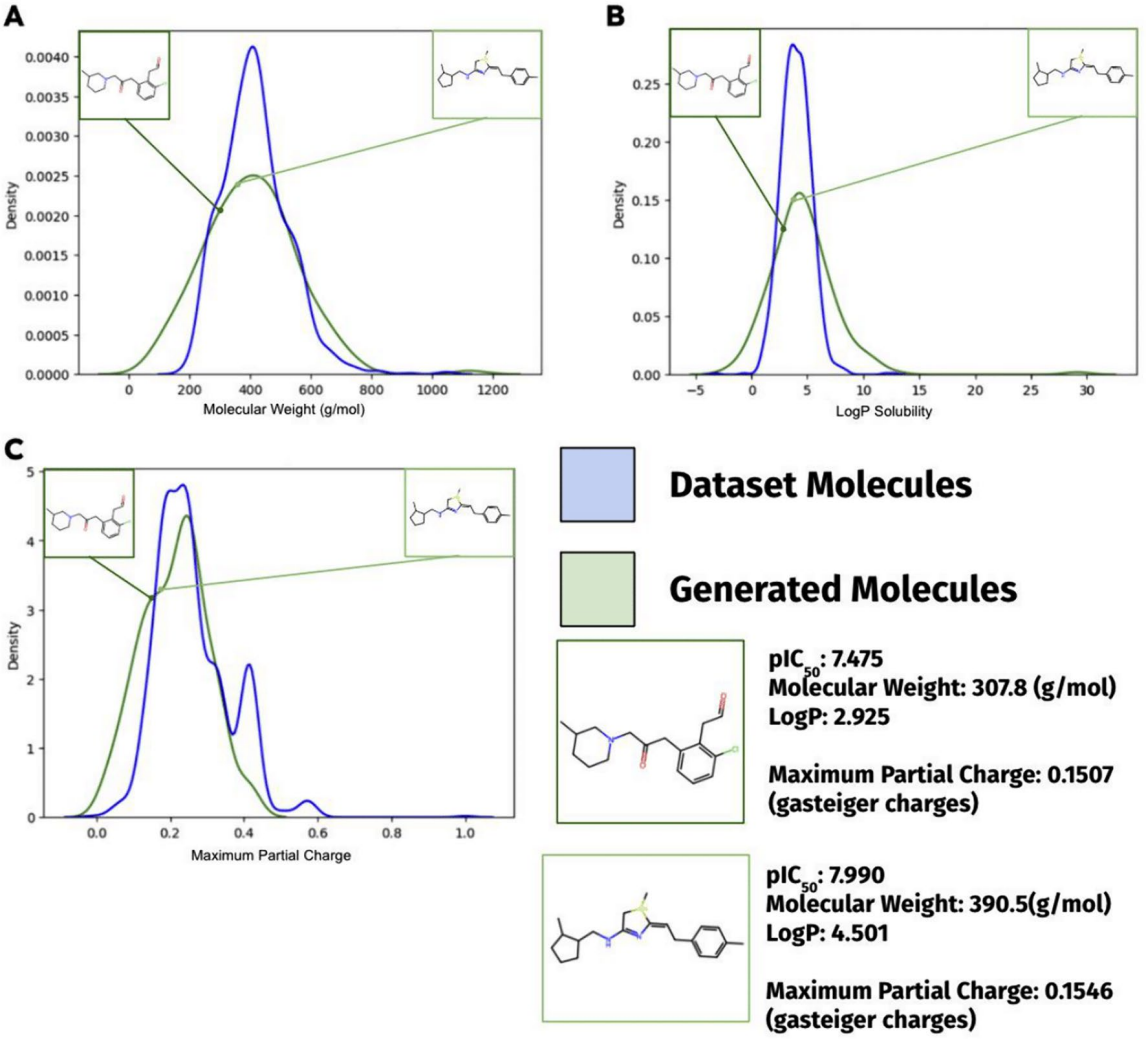
Sample of designed molecules*.* Sample of designed molecules are shown in the context of the property distributions of the dataset and the generated molecules. Both are novel molecules that do not exist in the dataset. The molecules are sampled post PPO, with a top p = 1 sampling strategy

A comparison to previous ML drug design models is shown (Table 1). The present study’s GPT model outperforms existing drug design models in designing effective, novel, and chemically valid molecules. Samples of designed molecules are also shown (Fig. 9). Most of the generated molecules exhibit desired properties such as high drug efficacy. Compared to the dataset (Fig. 8), on average the pIC_50_ value of the generated molecules is higher by 1.19, and the variance in pIC_50_ is much less in the generated set than in the training set. 99.2% of the molecules had pIC_50_ > 7 in the PPO training process. These results signify that NLP techniques and training schemas can also be transferred to the realm of molecule property optimization and drug design. This opens the door for other techniques, such as reinforcement learning from human feedback, to be used for LCMs. In addition, recent years have shown different approaches for multi-objective PPO algorithms applied to different fields [25, 26]; these algorithms’ implication on LCM can be further explored and compared to the common and straightforward “weighted sum” approach. Since there are many factors that need to be optimized in the drug development process before a drug candidate becomes an approved drug molecule, future studies can investigate the addition of optimizing for low toxicity or other properties using similar techniques as PPO.

**Table 1:** Comparising to previous drug design models

Metrics	Present study (%)	Pereira et al. (LSTM)	Abbasi et al. (GAN) (%)	Popova et al. (RNN) (%)
% High efficacy (pIC_50_ > 7)	**99.2**	–	59	42.2
% Validity	**100**	91.8%*	62.3	42.6
% Novelty	**100**	91.3%*	**100**	**100**

Preivous state-of-the-art drug design models (i.e., models that design drug-like molecules and optimizes for higher efficacy toward a certain drug target) and their metrics of molecule efficacy, validity, and novelty are shown. The best performance values for each metric are highlighted in bold. Pereira et al. (LSTM) [27] and Abbasi et al. (GAN) [3] are two state-of-the-art drug design models. Popova et al. (RNN) [28] has multiple configurations of the molecule design models, the efficacy maximizing model is used

*As the model from Pereira et al. (LSTM) contain bugs and cannot be currently run without errors, the metrics are default values reported from the paper, and the efficacy toward APP is unknown

The fact that my drug design model can design molecules with both high efficacy and 100% novelty signifies a promising way of using LCMs trained with NLP techniques and algorithms. In addition, notice when Open AI train their LLMs such as Instruct GPT and Chat GPT, reinforcement learning from human feedback (RLHF) is often involved [29]. To apply this training strategy for drug design, chemists can help, from rating, ranking, and prioritizing certain designed drug molecules, to designing better alternatives as examples and provide direct feedback for the model. RLHF is shown to be one of the best ways to incorporate human insights into a GPT model [30], which this strategy can potentially help the drug design model pick up chemists’ “intuitions’’ in designing drug molecules—patterns that are hard to quantify and hard to use quantitative metric to measure and optimize [30]. This will be the first time chemists can directly interact with the drug design model, “communicating” their experiences to the drug design model, increasing the model performance and potentially making machine designed molecules indistinguishable from human designed ones. However, although this proposed RLHF strategy of having chemists provide feedback to a large chemistry model to improve its performance is a promising one, this requires the involvement of multiple chemists, and the cost would be unattainable and thus infeasible for the present study.

## Conclusion

A three-step LLM training process from Natural Language Processing was successfully applied in the context of drug design for LCM. This is an unprecedented strategy for large chemistry models, consisting of reward modeling, supervised finetuning, and proximal policy optimization (or RL optimization). To begin, in the reward modeling step, using publicly available experimental dataset from BindingDB [13] for protein-molecule interactions, and by combining sequential molecule representation with numerical chemical descriptors, this strategy modeled drug efficacy 2.34 times better accuracy than previous sequential and molecule fingerprint or descriptor-based QSAR evaluation models. In addition, the Exmol was used for the efficacy evaluation model for the first time and proved capable in adding explainability to evaluation models, especially when given customized, specific molecule descriptors in future studies. Then, in the Supervised Finetuning step, my drug design model successfully learned to generate molecules similar to those from the dataset. Finally, my evaluation model and my drug design model were used in the PPO optimization loop, where the designed molecules are analyzed by my evaluation model for the drug designing LCM model to improve, where 99.2% of the designed molecules have high efficacy (pIC_50_ > 7) and all are valid and novel. This approach is the first time it is employed on a LCM. The ability for my drug design model in generating molecules with high efficacy and novelty signifies that NLP techniques and training schemas can also be transferred in the realm of molecule modeling and drug design.

Although the GPT-Neo [21] with 19 million parameters was used as the base model due to limitations in computational resources in this study, the exact same methodology can be used for finetuning larger base models with much more parameters. The investigation of larger and more recent LLM such as LLama2-7B [19] (7 billion parameters) can be explored in future studies, where ChemGPT’s [9] pretraining procedure can be used for learning the SELFIES molecule representation, and the methodology in this study can be used for applying the LCM to drug design. Implications and future studies on LCM on drug design include designing novel developmental drug candidates satisfying multiple constraints and making patented drugs accessible by generating similar alternative molecules, all of which have the potential to transform drug discovery.

Although this study focused on a case-study in generating highly effective molecules towards the APP target protein, the same methodology can be applied to transfer LCM for targeting a different protein using a different dataset, facilitating and speeding up the discovery of more potential drug molecules for treating different diseases. This LCM showed exciting potential for NLP techniques (and LLM in general) to be applied in drug design, an imperative stride towards progress in the realm of large scientific models and drug design.

## Data Availability

The author is currently unable to specify which data has been produced. The sources and code will be available (upon request to yeeeyee004@gmail.com). Model will be available at https://huggingface.co/Coconut104/EfficacyGPT-DrugDesign
